# APC/C-Cdh1-dependent anaphase and telophase progression during mitotic slippage

**DOI:** 10.1186/1747-1028-7-4

**Published:** 2012-02-09

**Authors:** Kazuhiro Toda, Kayoko Naito, Satoru Mase, Masaru Ueno, Masahiro Uritani, Ayumu Yamamoto, Takashi Ushimaru

**Affiliations:** 1 Faculty of Science, Shizuoka University, Shizuoka University, Shizuoka 422-8529, Japan; 2 Department of Molecular Biotechnology, Graduate School of Advanced Sciences of Matter, Hiroshima University, 1-3-1 Kagamiyama, Higashi-Hiroshima 739-8530, Japan

**Keywords:** Anaphase promoting complex/cyclosome (APC/C), Bub2, Cdh1, mitotic exit network (MEN), mitotic slippage, *Saccharomyces cerevisiae*, securin

## Abstract

**Background:**

The spindle assembly checkpoint (SAC) inhibits anaphase progression in the presence of insufficient kinetochore-microtubule attachments, but cells can eventually override mitotic arrest by a process known as mitotic slippage or adaptation. This is a problem for cancer chemotherapy using microtubule poisons.

**Results:**

Here we describe mitotic slippage in yeast *bub2Δ *mutant cells that are defective in the repression of precocious telophase onset (mitotic exit). Precocious activation of anaphase promoting complex/cyclosome (APC/C)-Cdh1 caused mitotic slippage in the presence of nocodazole, while the SAC was still active. APC/C-Cdh1, but not APC/C-Cdc20, triggered anaphase progression (securin degradation, separase-mediated cohesin cleavage, sister-chromatid separation and chromosome missegregation), in addition to telophase onset (mitotic exit), during mitotic slippage. This demonstrates that an inhibitory system not only of APC/C-Cdc20 but also of APC/C-Cdh1 is critical for accurate chromosome segregation in the presence of insufficient kinetochore-microtubule attachments.

**Conclusions:**

The sequential activation of APC/C-Cdc20 to APC/C-Cdh1 during mitosis is central to accurate mitosis. Precocious activation of APC/C-Cdh1 in metaphase (pre-anaphase) causes mitotic slippage in SAC-activated cells. For the prevention of mitotic slippage, concomitant inhibition of APC/C-Cdh1 may be effective for tumor therapy with mitotic spindle poisons in humans.

## Background

The anaphase-promoting complex/cyclosome (APC/C) is an E3 ubiquitin ligase that plays a major role in cell cycle control by targeting substrates for proteasomal degradation. The complex is activated by two WD40 activator proteins, Cdc20/Fizzy/Fzy or Cdh1/Fizzy-related/Fzr. This destruction is strictly ordered to ensure that cell cycle events are executed in a timely fashion [1-5]. Whereas APC/C-Cdc20 is activated at metaphase-anaphase transition, APC/C-Cdh1 is activated after APC/C-Cdc20 activation. In the budding yeast *Saccharomyces cerevisiae*, APC/C-Cdh1 is activated from telophase to late G1 phase [6,7]. The switch from APC/C-Cdc20 to APC/C-Cdh1 is regulated by multiple mechanisms [5,8-10]: Cyclin B-Cdk1 (cyclin-dependent kinase) inhibits Cdh1 activation in metaphase, but cyclin B degradation mediated by APC/C in late M phase reduces cyclin B-Cdk1 activity, leading to Cdh1 activation. In addition, APC/C-Cdh1 mediates Cdc20 degradation, thereby promoting switching from APC/C-Cdc20 to APC/C-Cdh1.

The spindle assembly checkpoint (SAC) ensures faithful chromosome segregation during cell division [11,12]. In the presence of insufficient kinetochore-microtubule attachments, the SAC inhibits anaphase onset by the inhibition of APC/C-Cdc20. The SAC recruits checkpoint proteins, including Mad1, Mad2, Bub1, BubR1 (Mad3 in yeast), Bub3 and Mps1, to unattached kinetochores. As a result, Mad2, BubR1 and Bub3 bind to and suppress APC/C-Cdc20 and form the mitotic checkpoint complex (MCC) [13]. Once all chromosomes have achieved proper kinetochore-microtubule attachment, checkpoint signaling ceases, which is called SAC deactivation or inactivation, and Mad2/BubR1/Bub3 are released from APC/C-Cdc20. It allows active APC/C-Cdc20 to drive cells into anaphase by inducing the degradation of securin and cyclin B. The degradation of securin permits sister-chromatid separation, and the destruction of cyclin B reduces Cdk1 activity. In contrast to the molecular mechanisms of the SAC activation, those of SAC deactivation are poorly understood [14,15].

Microtubule targeted drugs are of clinical importance in the successful treatment of a variety of human cancers because they activate the SAC and induce mitotic arrest that leads to apoptotic cell death [16]. However, in the continued presence of conditions that normally keep the SAC active, some cells escape from mitosis, resulting in tetraploid cells [16,17]. This phenomenon is termed mitotic slippage or adaptation. This process is largely responsible for the failure to efficiently block tumor progression. Mitotic slippage depends on progressive degradation of cyclin B, while the SAC is active, indicating that mitotic slippage occurs through the overriding of activated SAC signaling [18,19]. Mitotic exit occurs once cyclin B-Cdk1 activity has decreased below a critical threshold required to maintain a mitotic state [18]. In addition to cyclin B, other mitotic APC/C substrates, including securin, are also degraded during mitotic slippage, and a double knockdown of Cdc20 and Cdh1 prevents the degradation of APC/C substrates during mitotic slippage [20]. These findings indicate that APC/C is critical for mitotic slippage. However, which protein does mitotic slippage require, Cdc20 or Cdh1? Furthermore, how can APC/C be activated, although the SAC is active? The degradation of Cyclin A and NIMA-related kinase 2A (Nek2A) in early mitosis is dependent on APC/C-Cdc20, and this process is not inhibited by the SAC [21]. While the SAC-dependent substrate cyclin B requires Cdc20 for recruitment to APC/C, Nek2A can bind the APC/C in the absence of Cdc20 [22]. Thus, the SAC suppresses the degradation of most, but not all, substrates of APC/C-Cdc20. However, degradation of cyclin A and Nek2A does not trigger metaphase-anaphase transition and mitotic slippage. It is unclear how mitotic exit (telophase onset) can be initiated in metaphase-arrested cells during mitotic slippage; less attention has been paid to how anaphase is executed during mitotic slippage.

In budding yeast, mitotic slippage-like phenomena have been reported, but they are relatively ill-defined, as compared with mammalian cells, because the SAC status is obscure. It is important to determine the SAC status during mitotic slippage (and slippage-like phenomena), in order to distinguish mitotic slippage that overrides the activated SAC from events caused by SAC deactivation. Mitotic exit accompanied by securin degradation, sister-chromatid separation and nuclear division was found after treatment of the wild-type yeast cells with the microtubule depolymerizer benomyl but not with nocodazole [23]. It is unknown whether these phenomena found in the presence of benomyl are indeed mitotic slippage, because the SAC status has not been characterized. Interestingly, mitotic slippage (or slippage-like phenomena) is prominently observed in mutant cells deficient in the *budding uninhibited by benzimidazole (BUB) 2 *gene in the presence of nocodazole. Among BUB proteins, whereas Bub1 and Bub3 are components of the SAC, Bub2 is an inhibitor of the mitotic exit network (MEN) that promotes anaphase-telophase transition [8-10,24,25]. Although *bub2Δ *cells exhibit an intact SAC, they fail to arrest in metaphase and exit from mitosis in the presence of nocodazole, leading to cell death [24,26-28]. In addition, *bub2Δ *cells cannot effectively arrest at metaphase when the SAC is activated by *MAD2 *overexpression [23].

Nocodazole-treated *bub2Δ *cells exhibit securin degradation, sister-chromatid separation (indexes of anaphase progression) and rebudding (an index of telophase progression and mitotic exit) [24,26,27]. Thus, anaphase and telophase progression occurs in nocodazole-treated *bub2Δ *cells. A mutation in the MEN factor Tem1 suppresses *bub2Δ*-induced sister-chromatid segregation [28]. It suggests that precocious activation of the MEN causes mitotic slippage, but the molecular mechanism responsible is largely unknown. Although securin degradation and sister-chromatid separation are normally mediated by APC/C-Cdc20 at anaphase onset, these events found in nocodazole-treated *bub2Δ *cells are not repressed by a *cdc20-3 *mutation at a restrictive temperature [29]. These findings indicate that anaphase progression in nocodazole-treated *bub2Δ *cells occurs independently of APC/C-Cdc20. On the other hand, sister-chromatid separation in nocodazole-*bub2Δ *cells is suppressed by a lack of the APC/C core subunit Cdc26 [27], suggesting that APC/C itself is required for *bub2Δ*-mediated anaphase progression. Thus, the *bub2Δ *stain might be a useful model for mitotic slippage. We show herein that nocodazole-treated *bub2Δ *cells override the active SAC-mediated metaphase arrest and cause securin degradation, sister-chromatid separation and mitotic exit and that APC/C-Cdh1 is critical for mitotic slippage.

## Results

### Precocious activation of the MEN induces mitotic slippage

**Figure 1:**
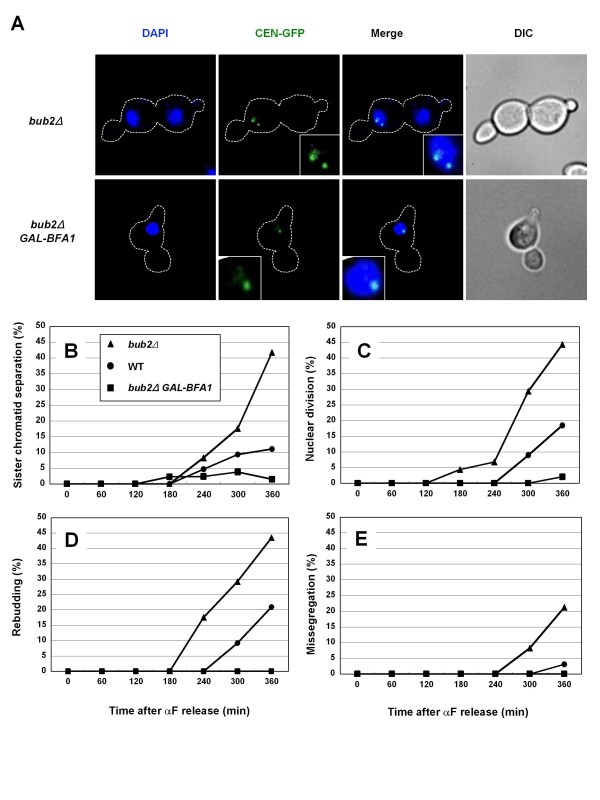
**Precocious activation of the MEN induces mitotic slippage**. (A-E) Exponentially growing cells of strains SCU396 (*CEN-GFP*) and SCU397 (*bub2Δ CEN-GFP*) harboring plasmids pSCU896 (pGAL1-BFA1) or pSCU134 (empty vector) were arrested in G1 phase by α-factor treatment (10 μg/ml) for 3 h and then released from α-factor into galactose-containing SGalR medium with nocodazole (15 μg/ml) (time 0). *BFA1 *was overexpressed under the control of the *GAL1 *promoter. Green fluorescent protein (GPF)-marked centromeres of chromosome IV were monitored for sister-chromatid separation (B) and chromosome missegregation (E). Bulk chromosome segregation (nuclear division), by means of staining with 4',6-diamidino-2-phenylindole (DAPI), (C) and rebudding (D) were also monitored. Representative cells at the 6 h time point are shown in (A).

We hypothesized that mitotic slippage in *bub2Δ *cells is caused by activation of the mitotic exit system in metaphase-arrested cells. To describe anaphase and telophase progression during mitotic slippage in *bub2Δ *cells, we followed sister-chromatid separation (an index of anaphase onset) using the lacO/lacI system [26] and new bud formation (rebudding; an index of telophase progression and mitotic exit). Cells were released from α-factor (G1 arrest) into a nocodazole-containing medium. The *bub2Δ *cells gradually overrode metaphase arrest and showed sister-chromatid separation and rebudding (Figures 1A, B and 1D), as described previously [24,26]. We observed frequent chromosome missegregation in the *bub2Δ *cells (Figures 1A, E). When the SAC is deactivated after establishment of proper kinetochore-microtubule attachment, chromosomes could be accurately segregated. In contrast, chromosome missegregation might frequently occur in cells during mitotic slippage, because under these conditions anaphase progression occurs even when there are improper/insufficient kinetochore-microtubule attachments. Chromosome missegregation observed here supports the occurrence of mitotic slippage in nocodazole-treated *bub2Δ *cells. In contrast, a smaller portion of wild-type cells showed sister-chromatid separation and rebudding under the same conditions. Thus, Bub2 is critical for the prevention of mitotic slippage.

Given the role of Bub2, it is most likely that precocious MEN activation occurs in *bub2Δ *cells, leading to mitotic slippage. To assess this idea, we examined whether overexpression of the MEN inhibitor Bfa1 cancels mitotic slippage in *bub2Δ *cells. This was indeed the case: sister-chromatid separation and rebudding was completely repressed by *BFA1 *overexpression (Figures 1A-C, *bub2Δ GAL-BFA1*). This demonstrated that MEN activation causes anaphase and telophase onset during mitotic slippage. It was also reported that a mutation in the MEN factor Tem1 suppresses *bub2Δ*-induced sister-chromatid segregation [28].

Because nuclear division (an index of anaphase progression) is dependent on spindle microtubules, it is inhibited when microtubules are completely abrogated. However, nuclear division in the *bub2Δ *cells treated with nocodazole (LKT Laboratories, Lot No. QJ1275) was identified, although no detectable microtubules were found in the indirect immunoflorescence assay (data not shown). This finding suggested that there were imperceptible microtubules causing nuclear division. However, it was noteworthy that the SAC was still active under these conditions (see below). These findings indicated that nocodazole continues to activate the SAC sufficiently and that the phenomena found in *bub2Δ *cells here are caused by mitotic slippage but not SAC deactivation/inactivation.

We also examined mitotic slippage of *bub2Δ *cells when the SAC gene *MAD2 *was overexpressed. *MAD2 *overexpression causes SAC activation-mediated metaphase arrest, but during a long-term treatment cells override metaphase arrest and cause cell proliferation, although profiles of sister-chromatid separation and nuclear division, chromosome missegregation during mitotic slippage were not described [23]. When *MAD2 *was overexpressed for 6 h, rebudding (mitotic exit) was frequently found in *bub2Δ *cells, as compared with wild-type cells (Additional file 1), which was consistent with the finding that cell proliferation was promoted by the *bub2Δ *mutation [23]. Furthermore, it was found that sister-chromosome segregation and nuclear division were also prominent in *bub2Δ *cells (Additional file 1). Thus, both nocodazole treatment and *MAD2 *overexpression similarly caused mitotic slippage in *bub2Δ *cells. In contrast, chromosome missegregation in *MAD2*-overexpressing *bub2Δ *cells was not detectable (data not shown), which was probably because in this case microtubules were intact and a proper kinetochore-microtubule attachment was established.

### APC/C-Cdh1 is critical for chromosome separation during mitotic slippage

**Figure 2:**
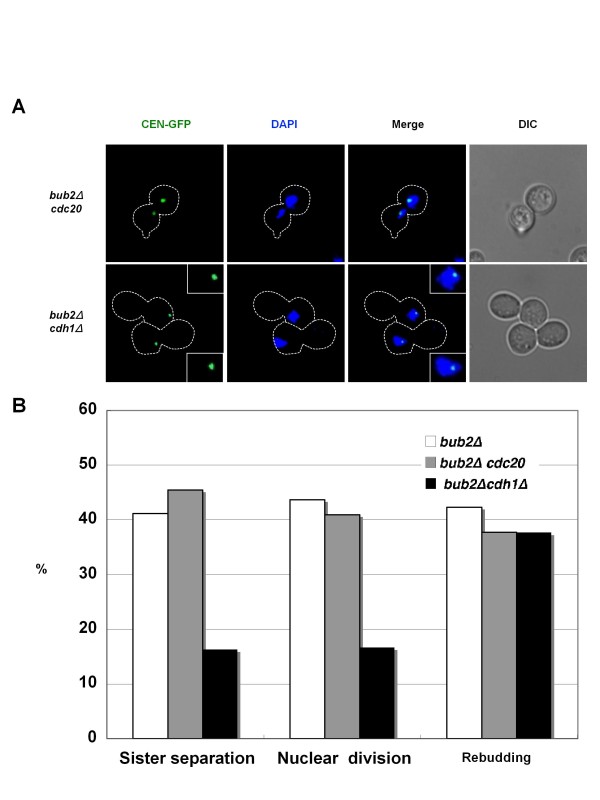
**APC/C-Cdh1 is critical for sister-chromatid separation during mitotic slippage**. (A, B) Cells of strains SCU399 (*bub2Δ CEN-GFP*), SCU410 (*bub2Δ CEN-GFP MET3-CDC20*) and SCU1336 (*bub2Δ CEN-GFP cdh1Δ*) were released from α-factor into the nocodazole-containing SGalR medium (time 0), as described in Figure 1. Representative cells at the 6 h time point are shown. Expression of *MET3 *promoter-driven *CDC20 *in strain SCU410 was shut off by the addition of methionine. The data for *bub2Δ *cells are taken from Figure 1, for comparison.

Sister-chromatid separation is triggered by APC/C-Cdc20 at normal anaphase onset, but APC/C-Cdc20 is suppressed by the SAC in nocodazole-treated cells. This suggests that APC/C-Cdc20 is not involved in mitotic slippage. In fact, it was reported that sister-chromatid separation in nocodazole-treated *bub2Δ *cells is not suppressed by a temperature sensitive *cdc20-3 *mutation [29]. We again found that Cdc20 depletion did not suppress sister-chromatid separation in the nocodazole-treated *bub2Δ *cells (Figure 2A, B). Rebudding was also not suppressed by Cdc20 depletion. This finding demonstrated that Cdc20 is not required for mitotic slippage. Consistently, it was reported that rebudding of nocodazole-treated *bub2Δ *cells was not suppressed by a temperature sensitive *cdc20-1 *mutation [30].

We suspected that precocious MEN activation in nocodazole-treated *bub2Δ *cells causes activation of APC/C-Cdh1, leading to mitotic slippage. Indeed, a lack of Cdh1 markedly repressed sister-chromatid separation and nuclear division in nocodazole-treated *bub2Δ *cells (Figure 2A, B). These observations clearly indicated that APC/C-Cdh1, but not APC/C-Cdc20, is responsible for MEN-mediated anaphase progression. In contrast, rebudding (mitotic exit) was not suppressed by *CDH1 *deletion, probably because the CDK inhibitor Sic1 induced by the MEN also contributes to the repression of CDK activity and is sufficient for mitotic exit in *cdh1Δ *cells, as in normal mitosis.

### APC/C-Cdh1-mediated securin degradation is required for sister-chromatid separation during mitotic slippage

**Figure 3:**
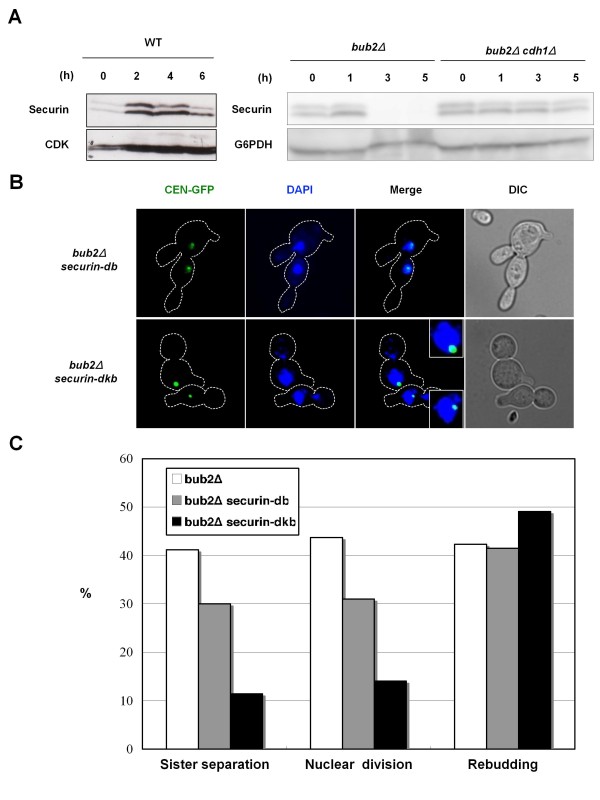
**APC/C-Cdh1-mediated securin degradation is required for sister-chromatid separation during mitotic slippage**. (A) Cells of strains SCU2755 (*PDS1-HA3*), SCU408 (*bub2Δ PDS1-HA3*) and SCU2834 (*bub2Δ cdh1Δ PDS1-HA3*) were released from α-factor into the nocodazole-containing medium (time 0), as described in Figure 1. HA-tagged securin (Pds1-HA3) was detected by western blotting analysis using an anti-HA antibody. CDK and glucose-6-phosphate dehydrogenase (G6PDH) were used as the loading controls. (B, C) Cells of strain SCU396 (*bub2Δ CEN-GFP*) with plasmids pSCU1212 (pGAL1-pds1-db) or pSCU1214 (pGAL1-pds1-dkb) were released from α-factor into nocodazole-containing SGalR medium (time 0), as described in Figure 1. Representative cells at the 6 h time point are shown. The data for *bub2Δ *cells are taken from Figure 1.

Sister-chromatid separation requires securin degradation. We examined securin degradation in nocodazole-treated *bub2Δ *cells. The yeast securin Pds1 is phosphorylated by CDK, and it was often detected as two bands in western blotting analysis [31,32]. Securin degradation was repressed in the presence of nocodazole by the SAC in wild-type cells, but it occurred in *bub2Δ *cells [27] (Figure 3A, WT and *bub2Δ*). Because sister-chromatid separation in nocodazole-treated *bub2Δ *cells was APC/C-Cdh1 dependent (Figure 2), it would be expected that APC/C-Cdh1 is required for securin degradation during mitotic slippage in nocodazole-treated *bub2Δ *cells. In the normal cell cycle, it was suggested that APC/C-Cdh1 mediates securin degradation after telophase onset (see "Discussion"). Indeed, securin degradation was largely suppressed by the loss of Cdh1 in *bub2Δ *cells (Figure 3A): *bub2Δ cdh1Δ *cells reproducibly had more securin at G1 phase (0 time point). This may result from the repression of securin degradation in the previous mitosis. Thereafter, securin levels were approximately equal between *bub2Δ *and *bub2 cdh1Δ *cells 1 hour after G1 release, and then securin degradation was obvious only in *bub2Δ *cells. Thus, APC/C-Cdh1 triggered securin degradation during mitotic slippage.

Next, we examined whether this securin degradation during mitotic slippage is required for sister-chromatid separation. APC/C-Cdh1 targets securin through D- and KEN boxes [33,34]. To test this, we ectopically expressed a non-degradable securin mutant devoid of both D- and KEN boxes (securin-dkb) [34]. Securin-dkb strongly repressed sister-chromatid separation (Figures 3B, C). In contrast, as expected, expression of a securin mutant lacking only the D-box (securin-db) repressed sister-chromatid separation less effectively. These findings demonstrated that APC/C-Cdh1-mediated securin degradation is a prerequisite for sister-chromatid separation during mitotic slippage in nocodazole-treated *bub2Δ *cells.

### Separase executes sister-chromatid separation and nucleolar segregation during mitotic slippage

**Figure 4:**
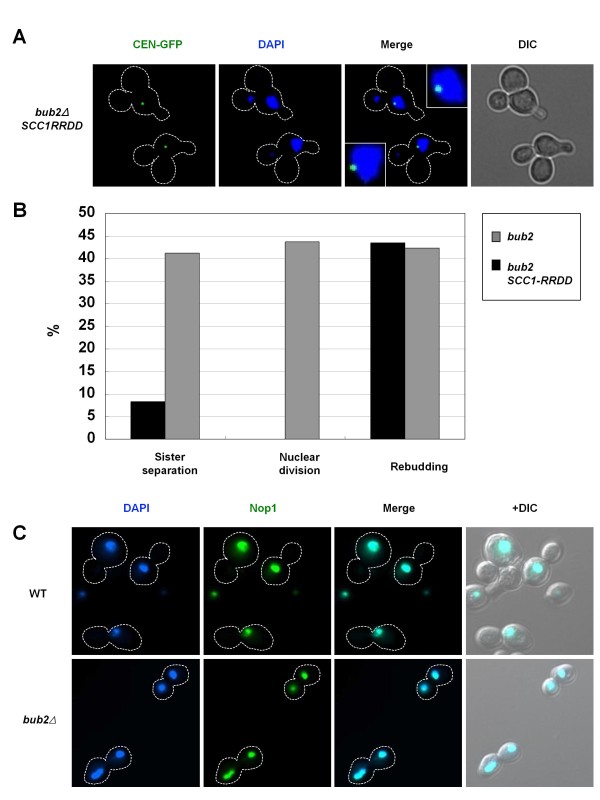
**Separase executes sister-chromatid separation and nucleolar segregation during mitotic slippage**. (A, B) Separase-mediated cohesin cleavage is required for sister-chromatid separation during mitotic slippage. Cells of a strain SCU404 (*bub2Δ CEN-GFP GAL-SCC1-RRDD*) were released from α-factor into the nocodazole-containing SGalR medium to induce the non-cleavable *SCC1-RRDD *(time 0), as described in Figure 1. Representative cells at the 6 h time point are shown. The data for *bub2Δ *cells are taken from Figure 1. (C) Nucleolar segregation during mitotic slippage. Cells of strains SCU893 (WT) and SCU151 (*bub2Δ*) strains with the plasmid pSCU740 (pGFP-NOP1) were released from α-factor into nocodazole-containing medium (time 0), as described in Figure 1. GFP-tagged Nop1 (nucleolar protein) and DAPI images are shown at the 6 h time point.

After APC/C-Cdc20-dependent securin degradation at normal anaphase onset, the liberated separase cleaves the subunit of cohesin Scc1, allowing sister-chromatid separation [35]. On the other hand, cohesin removal from chromosomes is also performed via another route; in higher eukaryotes, cohesins are largely dissociated from the chromosome arms in prophase in a separase-independent manner (called the prophase pathway) [36,37]. The prophase pathway has not yet been described in yeast, but recent reports suggested the occurrence of separase-independent cohesin removal and chromosome separation [38,39]. We examined whether separase-mediated Scc1 cleavage is required for sister-chromatid separation during mitotic slippage with the use of Scc1-RRDD, a non-cleavable mutant version of Scc1 [40]. Ectopic expression of Scc1-RRDD drastically suppressed sister-chromatid separation (Figures 4A, B). This clearly demonstrated that sister-chromatid separation requires separase-mediated Scc1 cleavage during mitotic slippage. Overall, APC/C-Cdh1 triggers securin degradation, separase liberation and then cohesin cleavage, causing sister-chromatid separation during mitotic slippage in nocodazole-treated *bub2Δ *cells.

In normal early anaphase, the liberated separase also causes nucleolar segregation in a manner independent of its protease activity [41-43]. The nucleolar segregation into mother and daughter cells was observed, together with nuclear division during mitotic slippage in the *bub2Δ *cells (Figure 4C). This indicates that protease-independent action of separase is also promoted during mitotic slippage.

### The SAC is active during mitotic slippage

**Figure 5:**
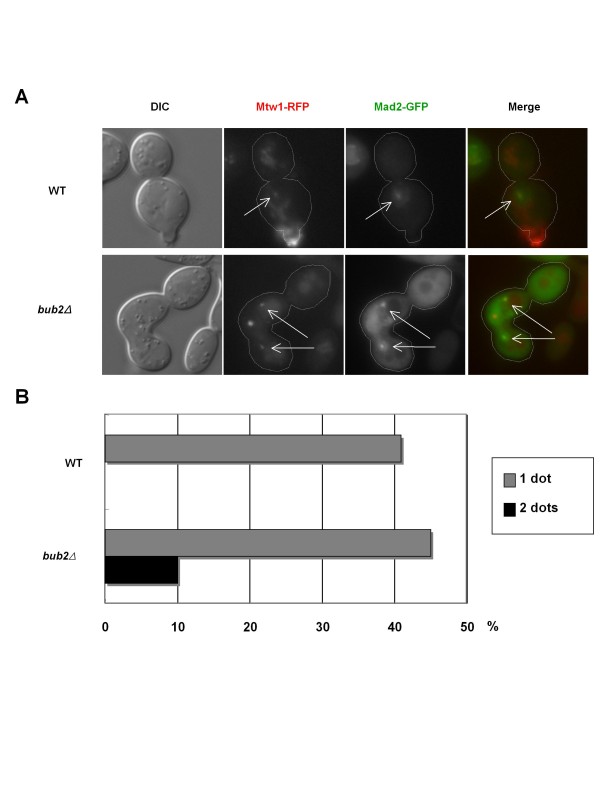
**The SAC is still active during mitotic slippage**. (A, B) Cells of strains SCU1337 (*MAD2-GFP*) and SCU1338 (*bub2Δ MAD2-GFP*) harboring the plasmid pSCU1701 (pMTW1-RFP) were released from α-factor into the nocodazole-containing medium (time 0), as described in Figure 1. Kinetochore localization of GFP-tagged Mad2 was monitored and counted after 6 h. White arrows indicate colocalized Mad2-GFP and Mtw1-RFP signals.

In mammalian cells, mitotic slippage occurs with SAC being still active and cells override the SAC-mediated metaphase arrest (see "Introduction"). To test whether the SAC is active in the present case, we examined kinetochore localization of Mad2, an index of SAC activation (Figure 5A). We observed GFP-tagged Mad2 signals on the kinetochores, which were marked by RFP (red fluorescent protein)-tagged Mtw1 (a kinetochore protein). We observed colocalization of Mad2 and Mtw1 signals in nocodazole-treated *bub2Δ *cells, as also observed in nocodazole-treated wild-type cells (Figures 5A, B), indicating that the SAC is still active in the *bub2Δ *cells, like in the wild-type cells. This demonstrates that SAC deactivation does not cause sister-chromatid separation, securin degradation and rebudding in the nocodazole-treated *bub2Δ *cells. Cell images were captured with single Z-axis sections using a microscope. Because the size of the kinetochore is considerably smaller than the cell, 100% of the signals of Mad2-GFP on the kinetochores were not detectable even if all Mad2 signals were localized on the kinetochores in all cells. It was noteworthy that both mother and daughter cells had one Mad2 dot signal on the kinetochore each in some *bub2Δ *cells (Figures 5A, B). Because kinetochore segregation to both mother and daughter cells indicates anaphase progression in these cells, these findings demonstrated that anaphase progression occurred with an active SAC in nocodazole-treated *bub2Δ *cells and that the cells overrides metaphase arrest by the activated SAC. Thus, precocious APC/C-Cdh1 activation overrides SAC-mediated metaphase arrest and causes mitotic slippage.

### Ectopic activation of Cdh1 causes mitotic slippage

**Figure 6:**
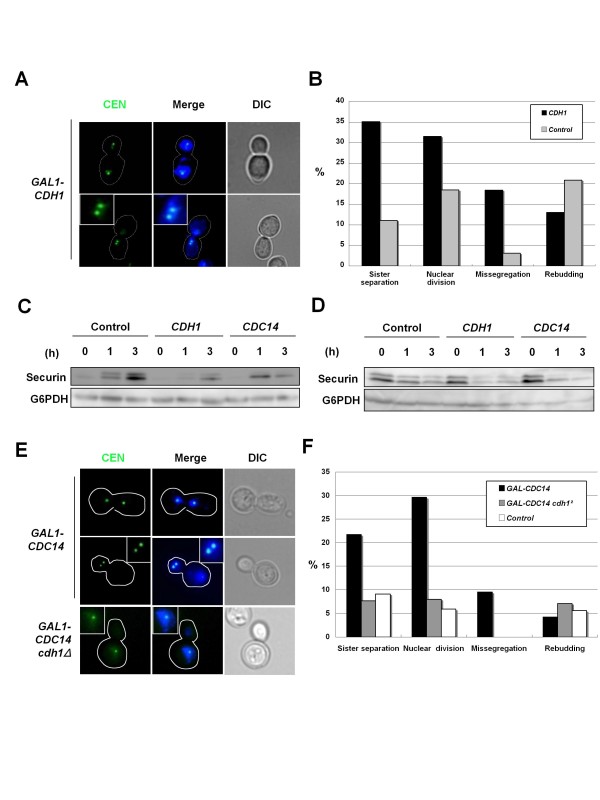
**Ectopic activation of Cdh1 causes mitotic slippage**. (A, B) Ectopic activation of Cdh1 causes mitotic slippage. Cells of a strain SCU398 (*CEN-GFP*) harboring plasmids pSCU878 (pGAL-CDH1) or pSCU145 (empty vector) were released from α-factor into nocodazole-containing SGalR medium (time 0), as described in Figure 1. Cell images and data were obtained after the 6 h time point. (C) Ectopic activation of Cdh1 and Cdc14 causes securin degradation. Cells of strain SCU2755 (*PDS1-HA3*) harboring plasmids pSCU145 (empty vector), pSCU1575 (pGAL-CDH1) or pSCU1576 (pGAL-CDC14) were released from α-factor into nocodazole-containing SGalR medium (time 0), as described in Figure 1. (D) The same cells as described in (C) were arrested using nocodazole and then galactose was added (time 0). (E, F) Ectopic activation of Cdc14 causes securin degradation in a manner dependent on Cdh1. Cells of strains SCU396 (*CEN-GFP*) and SCU1700 (*cdh1Δ CEN-GFP*) harboring the plasmid pSCU802 (pGAL-CDC14) were arrested in metaphase by nocodazole treatment for 3 h and then galactose was added for 3 h. Cells of the strain SCU396 (*CEN-GFP*) with plasmid pSCU134 (empty vector) were used as the control.

To test the idea that precocious activation of APC/C-Cdh1 causes mitotic slippage, we examined whether ectopic activation of Cdh1 similarly causes mitotic slippage. Cells were released from α-factor (G1 phase) to a medium containing nocodazole and galactose to overexpress *CDH1. CDH1 *overexpression brought about no securin accumulation, sister-chromatid separation or nuclear division with chromosome missegregation (Figures 6A-C). Rebudding was lowered by *CDH1 *overexpression under these conditions for an unknown reason (Figure 6B), whereas *CDH1 *overexpression in metaphase-arrested cells treated with nocodazole caused securin degradation and rebudding (Figure 6D and data not shown). Thus, ectopic activation of APC/C-Cdh1 caused mitotic slippage.

Cdc14 phosphatase, which antagonizes CDK, promotes APC/C-Cdh1 activation and mitotic exit in telophase [5,9,41]. *CDC14 *overexpression from G1 phase induced securin degradation (Figure 6C) but inhibited G1/S progression [44] (data not shown). Hence, we overexpressed *CDC14 *in metaphase-arrested cells treated with nocodazole. *CDC14 *overexpression promoted securin degradation and sister-chromatid separation (Figures 6D-F). However, both of these events were repressed in *cdh1Δ *cells (Figure 6E, F). These findings indicated that ectopic activation of Cdc14 causes mitotic slippage via APC/C-Cdh1. Consistent with the previous report that *CDC14 *overexpression promotes mitotic exit but represses budding in the next S phase, because of the counteraction of Cdc14 against CDK-mediated phosphorylation [44], no promotion of rebudding in the next S phase by *CDC14 *overexpression was observed (Figure 6F). Overall, these findings in Cdh1- and Cdc14-overexpressing cells supported the notion that precocious activation of APC/C-Cdh1 in pre-anaphase triggers mitotic slippage.

## Discussion

### Precocious activation of APC/C-Cdh1 in metaphase causes mitotic slippage

**Figure 7:**
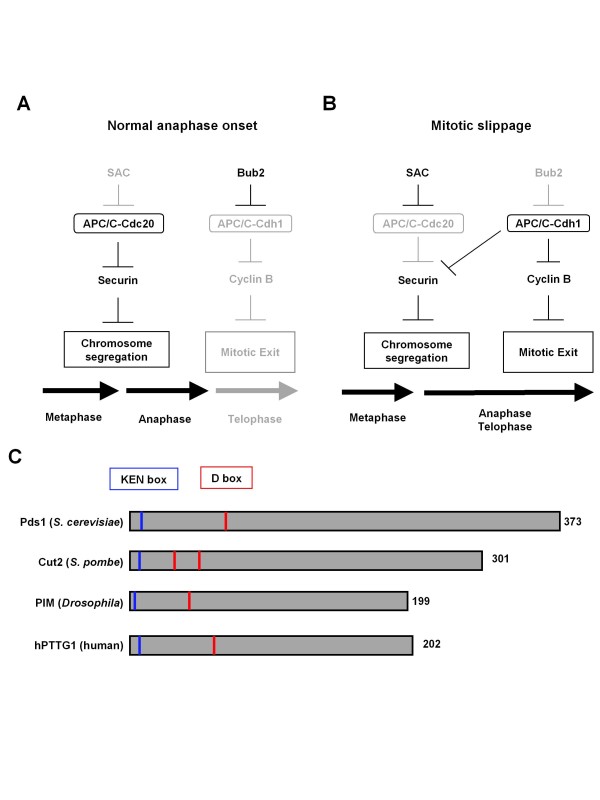
**A model for APC/C-Cdh1-mediated anaphase progression**. (A) APC/C-Cdc20-mediated anaphase progression in normal cell cycle. (B) APC/C-Cdh1-mediated anaphase and telophase progression during mitotic slippage. When proper kinetochore-microtubule attachments are not established, the SAC inhibits APC/C-Cdc20-mediated anaphase onset. However, loss (*bub2Δ*) or impairment of Bub2 function causes APC/C-Cdh1 triggered anaphase progression and telophase onset (mitotic exit). For details, see the text. (C) Schematic representation of securin proteins in *S. cerevisiae *(Pds1), *Schizosaccharomyces pombe *(Cut2), *Drosophila *(PIM) and Human (hPTTG). Blue and red lines indicate D- and KEN boxes.

While APC/C-Cdc20 is activated at metaphase-anaphase transition, APC/C-Cdh1 is activated at anaphase-telophase transition [6,7] (Figure 7A). The switch from APC/C-Cdc20 to APC/C-Cdh1 is regulated by multiple mechanisms [5,8-10]. This sequential activation is thought to be the heart of accurate mitosis, but this notion has not yet been fully tested. This study showed that precocious activation of APC/C-Cdh1 in metaphase (pre-anaphase) caused mitotic slippage in nocodazole-treated cells and that APC/C-Cdh1, instead of APC/C-Cdc20, could trigger anaphase progression, in addition to telophase progression (Figure 7B).

APC/C-Cdh1-mediated anaphase progression during mitotic slippage had two prominent features. First, APC/C-Cdh1-mediated anaphase progression brought about chromosome missegregation, because APC/C-Cdh1 is not inhibited by the SAC in the presence of inappropriate kinetochore-microtubule attachments; therefore, APC/C-Cdh1-mediated securin degradation results in chromosome missegregation. This demonstrated that an inhibitory system not only of APC/C-Cdc20 but also of APC/C-Cdh1 is critical for accurate chromosome segregation in the presence of insufficient kinetochore-microtubule attachments.

Second, APC/C-Cdh1 simultaneously starts anaphase and telophase from metaphase. APC/C-Cdc20 recognizes the D-box of a relatively limited umber of targets (the important targets are only cyclin Clb5 and securin Pds1 in budding yeast) [45], whereas APC/C-Cdh1 recognizes various motifs on numerous targets (A-, O-, CRY and GxEN boxes, in addition to D- and KEN boxes) [2,46-48]. Namely, APC/C-Cdh1 could target substrates for APC/C-Cdc20, which allowed simultaneous onsets of anaphase and telophase. APC/C-Cdh1 targets securin Pds1 in a manner dependent on D- and KEN boxes in vitro [33,34], and ectopically expressed Pds1 in G1 phase was degraded in a manner dependent on APC/C-Cdh1 [49]. These findings suggested that APC/C-Cdh1 mediates securin degradation from telophase to G1 phase in vivo. If APC/C-Cdh1 becomes activated abnormally in metaphase, it can target securin, leading to sister-chromatid separation (Figure 7B). This study clarifies these abnormal aspects of precocious APC/C-Cdh1 activation in metaphase cells and emphasizes that sequential activation of APC/C-Cdc20-to-APC/C-Cdh1 is critical for mitosis.

Deregulation of APC/C-Cdh1 in other cell phases brings about different outputs. *CDH1 *overexpression in asynchronized cells leads to elongated buds, G2 phase arrest, and 4C DNA content in some cells [6,7,50]. Precocious activation of APC/C-Cdh1 in G2 phase targets proteins that are required for separation of the spindle pole body (SPB, yeast centrosome), the BimC family kinesins Cin8/Eg5 and Kip1 and the interpolar microtubule midzone protein Ase1 [51,52]. Thus, deregulation of Cdh1 activity compromises genome transmission in various ways and timely activation and inactivation of APC/C-Cdh1 are pivotal for accurate genome transmission.

### APC/C-Cdh1-mediated mitotic slippage in other organisms

In fission yeast, the septation initiation network (SIN), a signaling pathway homologous to the MEN, coordinates mitosis and cytokinesis [8,25,53,54]. Cdc16 (Bub2 ortholog) acts as a negative factor of the SIN, and *cdc16 *mutant cells undergo cytokinesis in the presence of the microtubule destabilizer thiabendazole [55-57]. This suggests that SIN-mediated APC/C-Cdh1/Ste9 activation causes mitotic slippage. In addition, the fission yeast securin Cut2 also has D- and KEN boxes (Figure 7C). We postulate that APC/C-Cdh1/Ste9-mediated securin degradation and sister-chromatid separation is promoted during mitotic slippage in fission yeast.

In mammalian mitosis, APC/C-Cdc20 and APC/C-Cdh1 are sequentially activated [3,5,58]. Mitotic slippage depends on progressive degradation of cyclin B with the SAC active [18,19]. This suggests that APC/C-Cdh1, but not APC/C-Cdc20, is also involved in mitotic slippage in mammalian cells. Cdc20 and Cdh1 target securin in a manner dependent on D/KEN-boxes [59,60] (see Figure 7C). This suggests that precocious activation of APC/C-Cdh1 similarly causes securin degradation and sister-chromatid separation during mitotic slippage in mammalian cells. In fact, deregulation of Cdh1 in pre-anaphase results in premature securin degradation and sister-chromatid separation [59,61]. The present study predicts that for prevention of mitotic slippage, concomitant inhibition of APC/C-Cdh1 may be effective for tumor therapy with mitotic spindle poisons in humans.

## Conclusions

The sequential activation of APC/C-Cdc20-to-APC/C-Cdh1 during mitosis is critical for accurate mitosis. Precocious activation of APC/C-Cdh1 in metaphase (pre-anaphase) causes mitotic slippage in microtubule poison-treated cells. For prevention of mitotic slippage, concomitant inhibition of APC/C-Cdh1 may be effective for tumor therapy with mitotic spindle poisons in human.

## Methods

### Strains, plasmids, media and materials

*S. cerevisiae *strains and plasmids used are listed in Tables 1 and 2. Glucose-containing YPAD (YPD containing 0.01% adenine) and synthetic minimal medium (SD) complemented with the appropriate nutrients for plasmid maintenance were prepared using standard methods. SGalR and SRGly were identical to SD except that they contained 1% galactose plus 1% raffinose, and 2% galactose plus 3% glycerol instead of 2% glucose, respectively. Nocodazole and α-factor were purchased from LKT Laboratories (St. Paul, MN, USA) and Genenet (Fukuoka, Japan), respectively.

**Table 1:** Yeast strains used in this study

Name (Alias)	Description (Source)
SCU15 (W303a)	*Mata ura3 his3 leu2 trp1 ade2 can1 *(lab stock)
SCU151 (*bub2Δ*)	SCU893 *bub2::hphMX4 *(this study)
SCU396 (*CEN-GFP*)	SCU893 *his3::GFP12-LacI12-NLS::HIS3 trp1::LacOx256-TRP1 *(this study)
SCU397 (*bub2Δ CEN-GFP*)	SCU396 *bub2::loxP *(this study)
SCU398 (*CEN-GFP*)	SCU893 *ura3::tetO2x112::URA3 leu2::tetR-GFP-NLS::LEU2 *(this study)
SCU399 (*bub2Δ CEN-GFP*)	SCU398 *bub2::hphMX *(this study)
SCU404 (*bub2Δ GAL-SCC1-RRDD CEN-GFP*)	SCU397 *leu2::GAL1-SCC1-R180D/R268D-HA3::LEU2 *(this study)
SCU408 (*bub2Δ PDS1-HA3*)	SCU151 *PDS1-HA3::URA3 *(this study)
SCU410 (*bub2Δ MET3-CDC20 CEN-GFP*)	SCU399 *MET3-CDC20::TRP1 *(this study)
SCU893 (*bar1Δ*)	SCU15 *bar1::hisG *(U. Surana)
SCU1226 (*cdh1Δ CEN-GFP*)	SCU15 *ura3::tetO::URA3 leu2::tetR::LEU2 cdh1::HIS3 *[63]
SCU1228 (*cdh1Δ*)	SCU15 *cdh1::kanR *[63]
SCU1336 (*bub2Δ cdh1Δ CEN-GFP*)	SCU1226 *bub2::kanMX *(this study)
SCU1337 (*MAD2-GFP*)	SCU893 *mad2::kanM*X [pMAD2-GFP] (this study)
SCU1338 (*bub2Δ MAD2-GFP*)	SCU151 *mad2::kanMX *[pMAD2-GFP] (this study)
SCU1700 (*cdh1Δ CEN-GFP*)	SCU1228 *trp1::LacOx256:TRP1 his3::HIS3p-GFP13-LacI12NLS::HIS3 *(this study)
SCU2755 (*PDS1-HA3*)	SCU893 *pds1::PDS1-HA3::URA3 *(this study)
SCU2834 (*bub2Δ cdh1Δ PDS1-HA3*)	SCU1336 *ura3 pds1::PDS1-HA3::URA3 *(this study)

**Table 2:** Plasmids used in this study

Name (Alias)	Description (Source)
pSCU134 (p416GAL1)	*GAL1 URA3 CEN *[64]
pSCU145 (pRS414)	*TRP1 CEN *[65]
pSCU563 (pLacOx256-LEU2)	*lacOx256 LEU2 *integrative [26]
pSCU564 (pGFP12-LacI12-NLS)	*CUP1pro-GFP12*-*LacI12-NLS HIS3 *integrative [26]
pSCU683 (ptetR-GFP)	*NLS-tetR-GFP LEU2 *integrative [66]
pSCU710 (ptetO2x112)	*tetO2x112 URA3 *integrative [66]
pSCU740 (pNOP1-GFP)	*NOP1-HA3GFP URA3 CEN *(this study)
pSCU784 (pPDS1-HA3)	*PDS1-HA3 URA3 *integrative [31]
pSCU802 (pGAL1-CDC14)	*GAL-CDC14-His6 URA3 CEN *[67]
pSCU816 (pGAL-SCC1-RRDD)	*GAL-SCC1-R180D/R268D-HA3 LEU2 *integrative [40]
pSCU878 (pGAL-CDH1)	*GAL-CDH1-GFP TRP1 CEN *[68]
pSCU896 (pGAL1-BFA1)	*GAL1-BFA1 URA3 CEN *[69]
pSCU973 (pMAD2-GFP)	*MAD2-GFP URA3 CEN *[70]
pSCU985 (YIp22-MET3-CDC20)	*MET3-CDC20 TRP1 *integrative (F. Uhlmann)
pSCU1212 (p416GAL1-pds1-db)	*GAL1-PDS1 *with mutated D-box *URA3 CEN *(this study)
pSCU1214 (p416GAL1-pds1-dkb)	*GAL1-PDS1 *with mutated D/KEN-box *URA3 CEN *(this study)
pSCU1550 (pGAL1-MAD2)	*GAL1-MAD2-His6HAZZ 2 μ URA3 *[71]
pSCU1575 (pGAL1-CDH1)	*GAL1-CDH1-TAP 2 μ TRP1 *(this study)
pSCU1576 (pGAL1-CDC14)	*GAL1-CDC14-His6HAZZ 2 μ TRP1 *(this study)
pSCU1701 (pMTW1-RFP)	*MTW1-DsRed.T4 CEN LEU2 *(this study)

### Microscope observations

Except for Mad2-GFP-expressing cells, cells expressing GFP-tagged proteins were fixed with 70% ethanol for 30 sec. After washing with distilled water, cells were stained with 4',6-diamidino-2-phenylindole (DAPI) at 1 μg/ml for 15 min. For detection of weak Mad2-GFP signals, cells were not fixed with ethanol. Washed cells were viewed using an Olympus IX71-23FL/S microscope (100× objective) and a cooled charge-couple device (CCD) camera (ORCA-ER-1, Hamamatsu Photonics) connected to a Scanalytics Image Processor LuminaVision (Mitani Corp., Tokyo, Japan). For Figures 4C and 5A and Additional file 1A, a Carl Zeiss Axio Imager M1 microscope with a cooled CCD camera (Carl Zeiss AxioCam MRm) was used.

### Western blotting analysis

Western blotting was performed as described previously [62] using an anti-hemagglutinin (HA) antibody (16B12, BAbCo), anti-cyclin dependent kinase (CDK) antibody (Santa Cruz), and anti-glucose-6-phosphate dehydrogenase (G6PDH) antibody (Sigma). Femtogrow chemiluminescent substrate (Michigan Diagnostics) for horseradish peroxidase (HRP) and Can Get Signals (Toyobo, Japan) as an immunoreaction enhancer solution were used. Chemiluminescent signals were detected using an LAS3000 mini (Fuji).

## Competing interests

The authors declare that they have no competing interests.

## Authors' contributions

KT conducted the vast majority of the experiments described in this manuscript. KN performed the experiments demonstrating securin degradation in mutant strains and kinetochore localization of Mad2. SM performed the experiments demonstrating mitotic slippage in *MAD2*-overexpressing cells. TU designed the study, supervised the work, and wrote the manuscript. M. Ueno, M. Uritani and AY contributed to design of experiments. All authors approved the final manuscript.

## Authors' information

Present address of M. Ueno: Department of Molecular Biotechnology, Graduate School of Advanced Sciences of Matter, Hiroshima University, 1-3-1 Kagamiyama, Higashi-Hiroshima 739-8530, Japan

## Supplementary Material

Additional file 1 **Mitotic slippage of *MAD2*-overexpressing *bub2Δ *cells**. (A, B) Cells of strains SCU396 (CEN-GFP) and SCU397 (bub2Δ CEN-GFP) harboring plasmid pSCU1550 (pGAL-MAD2) were released from α-factor into the nocodazole-containing medium (time 0), as described in Figure 1. Kinetochore localization of Mad2-GFP was monitored and counted after 6 h. White arrows indicate colocalized Mad2-GFP and Mtw1-RFP signals. Click here for file
