# Supplementary figures and images for: APC/C-Cdh1-dependent anaphase and telophase progression during mitotic slippage
Source: Cell Div. 2012 Feb 9;7:4. doi: 10.1186/1747-1028-7-4 (PMC3305350; doi:10.1186/1747-1028-7-4)

## Slide 1
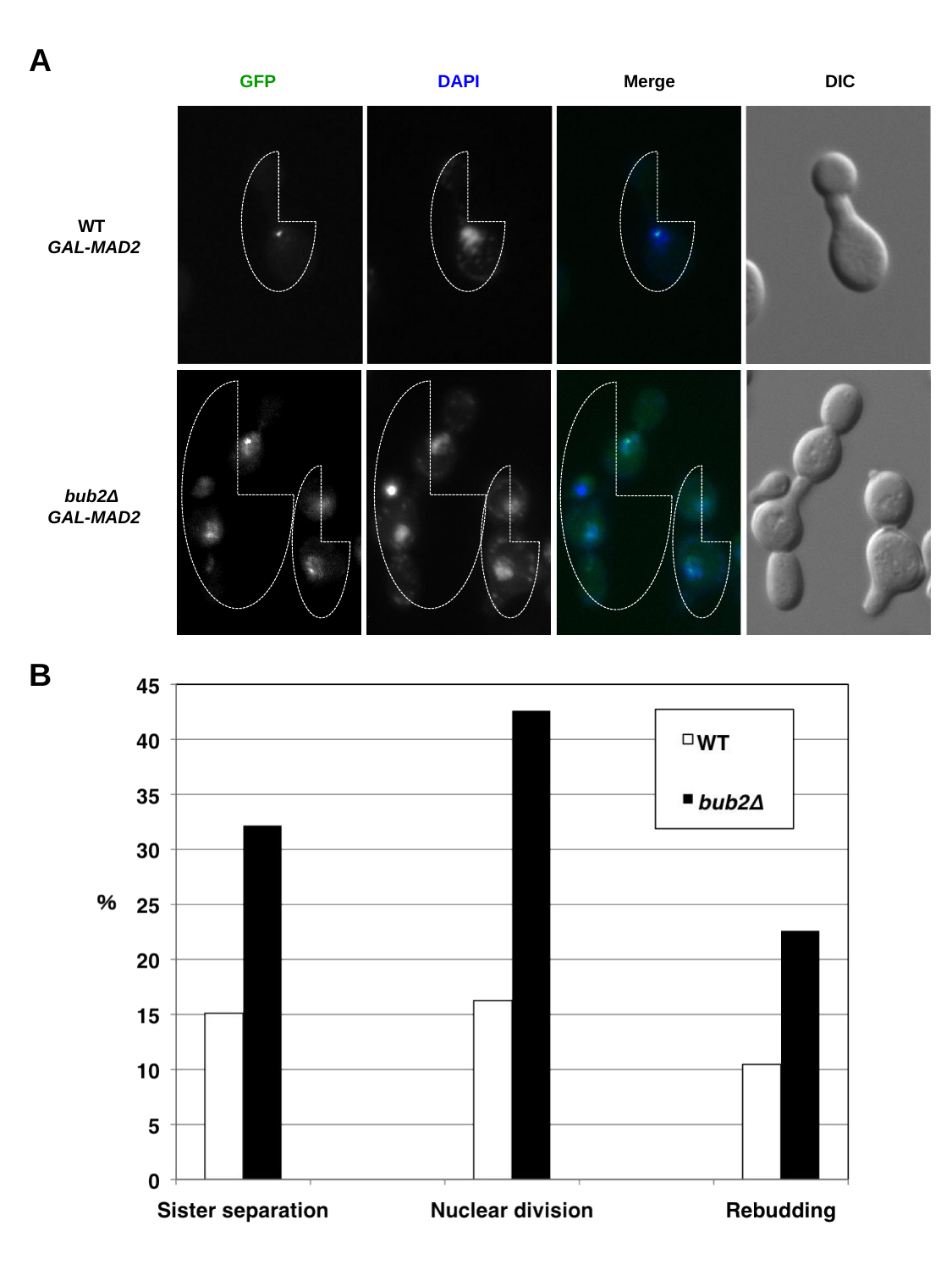

A
GFP
DAPI
Merge
DIC
WT
 GAL-MAD2
bub2Δ
 GAL-MAD2
B

Supplement: Additional file 1 — Mitotic slippage of MAD2-overexpressing bub2Δ cells. (A, B) Cells of strains SCU396 (CEN-GFP) and SCU397 (bub2Δ CEN-GFP) harboring plasmid pSCU1550 (pGAL-MAD2) were released from α-factor into the nocodazole-containing medium (time 0), as described in Figure 1. Kinetochore localization of Mad2-GFP was monitored and counted after 6 h. White arrows indicate colocalized Mad2-GFP and Mtw1-RFP signals. [file 1747-1028-7-4-S1.PPT]
